# Cancer-associated fibroblasts, tumor and radiotherapy: interactions in the tumor micro-environment

**DOI:** 10.1186/s13046-024-03251-0

**Published:** 2024-12-19

**Authors:** Kris T. P. M. Raaijmakers, Gosse J. Adema, Johan Bussink, Marleen Ansems

**Affiliations:** Radiotherapy and OncoImmunology Laboratory, Department of Radiation Oncology, Radboud University Medical Center, Nijmegen, the Netherlands

**Keywords:** Cancer-associated fibroblasts, Tumor micro-environment, Therapy resistance, Radioresistance

## Abstract

Cancer-associated fibroblasts (CAFs) represent a group of genotypically non-malignant stromal cells in the tumor micro-environment (TME) of solid tumors that encompasses up to 80% of the tumor volume. Even though the phenotypic diversity and plasticity of CAFs complicates research, it is well-established that CAFs can affect many aspects of tumor progression, including growth, invasion and therapy resistance. Although anti-tumorigenic properties of CAFs have been reported, the majority of research demonstrates a pro-tumorigenic role for CAFs via (in)direct signaling to cancer cells, immunomodulation and extracellular matrix (ECM) remodeling. Following harsh therapeutic approaches such as radio- and/or chemotherapy, CAFs do not die but rather become senescent. Upon conversion towards senescence, many pro-tumorigenic characteristics of CAFs are preserved or even amplified. Senescent CAFs continue to promote tumor cell therapy resistance, modulate the ECM, stimulate epithelial-to-mesenchymal transition (EMT) and induce immunosuppression. Consequently, CAFs play a significant role in tumor cell survival, relapse and potentially malignant transformation of surviving cancer cells following therapy. Modulating CAF functioning in the TME therefore is a critical area of research. Proposed strategies to enhance therapeutic efficacy include reverting senescent CAFs towards a quiescent phenotype or selectively targeting (non-)senescent CAFs. In this review, we discuss CAF functioning in the TME before and during therapy, with a strong focus on radiotherapy. In the future, CAF functioning in the therapeutic TME should be taken into account when designing treatment plans and new therapeutic approaches.

## Introduction

The tumor stroma plays a significant role in virtually all aspects of the growth of solid tumors, including invasion and metastatic capacity, as well as resistance to treatment. The majority of the tumor stroma consists of cancer-associated fibroblasts (CAFs) [1, 2]. Whereas normal fibroblasts are in a quiescent state during tissue homeostasis, CAFs assume a permanently activated phenotype and are both functionally and epigenetically different than normal fibroblasts [3, 4]. In addition, CAFs display a high degree of intrinsic heterogeneity; CAFs can derive from several cell types, including epithelial cells, resident fibroblasts, and mesenchymal stem cells [5].

In recent years, it is increasingly recognized that the intra-tumoral CAF population consists of different subsets exerting different functions [6–8]. Although no consensus on the exact number of subsets has been reached, two subtypes appear in the vast majority of analyses; αSMA^+^ myofibroblastic CAFs (myCAFs) that are strongly involved in ECM deposition, and αSMA^−^ inflammatory CAFs (iCAFs), that are characterized by an increased production of immunomodulating cytokines [9].

The exact role of CAFs in tumor initiation, progression and treatment resistance remains elusive. Cancer cells can be affected by CAFs via the CAF secretome, via cell–cell contact, or via indirect interaction between CAFs and cancer cells via the extracellular matrix (ECM) [10–13]. Both pro-tumorigenic as well as anti-tumorigenic properties have been ascribed to CAFs [8, 14–16], and different roles in tumor initiation and tumor progression have been suggested. Consequences of CAF functioning in the TME include pro-survival signaling towards tumor cells, remodeling of the ECM, immunomodulation and therapy resistance [10].

The composition of the CAF landscape and its functioning in different types of cancers and under different types of treatment are incompletely understood. In addition, several studies show that CAF subsets are not static. Instead, CAFs display subset plasticity, facilitated by epigenetic modulation, that can also be induced by therapeutic intervention [4, 17–19]. The characterization and functioning of CAF subsets is still an ambiguous topic that requires further research.

This review focuses on the role of CAFs in the TME during treatment with a special focus on radiotherapy, and the effect of CAFs on tumor progression and treatment resistance.

## The role of CAFs in the tumor microenvironment

CAFs have been shown to exert an anti-tumorigenic function; for example by providing a physical barrier for the tumor, mechanically restraining tumor growth [14, 15]. However, the majority of research demonstrates a pro-tumorigenic role for CAFs in both the non-treated and the post-therapy micro-environment. In addition, it has been reported that modulation of CAF functioning prior to and during treatment can impact treatment efficacy.

### Cell proliferation and pro-survival signaling

Signaling between cancer cells and CAFs occurs bidirectionally. Conditioned medium from CAFs has been shown to stimulate cancer cell proliferation [20, 21] and vice versa [22, 23], suggesting the presence of a positive feedback loop stimulating the proliferation of both CAFs and cancer cells. The CAF secretome induces the proliferation of cancer cells via the secretion of cytokines, such as CXCL-12, EGF, HGF [22, 24–26]; with energy-rich metabolites, such as ketones, lactate and glutamine [27–31]; and via exosomes [32, 33]. Apart from proliferation, CAFs also stimulate cancer cell survival during both chemo- and radiotherapy through exosomally delivered molecules or through direct signaling, for instance via IGF1/2, CXCL12 and β-hydroxybutyrate [34–36].

### Matrix reorganization and epithelial-mesenchymal transition (EMT) induction

The matrix-remodeling capacity of CAFs consists of both matrix deposition and degradation: CAFs strongly contribute to ECM formation by the deposition of matrix molecules such as fibronectin and collagen, leading to an increased stiffness of the TME [37–40]. In addition, the increased integrin expression on CAFs induces the bundling of existing collagen fibers or remodeling of the structure of collagen and fibronectin fibers [8, 41], further contributing to ECM stiffness. The resulting altered tissue microarchitecture leads to elevated physical stress-induced vascular compression and increased interstitial fluid pressure, which prevents drugs and immune cells from reaching the tumor. In addition, the increased mechanical tension can induce cancer cell EMT via the mechanosensitive YAP/TAZ and/or TWIST1 signaling axis, leading to increased invasive capacity, dissemination and therapy resistance of tumor cells [42–44].

Additionally, it has been shown that CAF-deposited matrix components contribute to the compression of vessels, which subsequently could lead to intratumoral hypoxia [45, 46]. The resulting activation of HIF-1α and the corresponding transcriptional program contribute to tumor progression by both stimulating cancer cell survival and proliferation and to tumor invasiveness via the induction of EMT. CAFs can thus contribute to several changes in the TME that lead to tumor progression via the combined induction of ECM stiffness, hypoxia and EMT [47–49].

### Angiogenesis and immunosuppression

CAFs stimulate angiogenesis via ECM reorganization and the resulting activation of the mechanosensitive transcription factor YAP [50, 51], via the secretion of pro-angiogenic compounds such as VEGF and CXCL12 [26, 52], or by expressing galactin-1 and podoplanin [53]. Alternatively, the compression of blood vessels due to CAF-mediated matrix deposition can lead to hypoxia, which can result in HIF-1α activation, angiogenesis, CAF activation and increased expression of the immunosuppressive PD-L1 [51, 54–56]. Tumor vasculature is not only of high importance for oxygen and nutrient availability, but also for the functioning of the immune system. CAFs influence the immune system by secreting a considerable amount of immunomodulatory cytokines and chemokines, such as IL-6, IL-10 and TGFβ [3, 57, 58]. CAFs are shown to induce the conversion of immune cells towards a pro-tumorigenic phenotype [59]. In addition, CAFs can restrict immune cell recruitment, either via the secretion of specific chemokines [12, 60] or by impairing access of immune cells to the TME via reorganization of the ECM [19, 61, 62]. Additionally, CAF-mediated activation of HIF-1α can lead to the upregulation of the immune-inhibitory PD-L1 on both immune cells and tumor cells [53]. CAFs affect the functioning of nearly all immune cells, as reviewed in Desbois et al. [63], Kennel et al. [64] and Maia et al. [65].

### Recruitment of new CAFs

CAFs can also induce the conversion of normal fibroblasts into CAFs, or contribute to sustaining the phenotype of other CAFs. High contractile forces in the TME induced by CAFs promote the activation of quiescent fibroblasts towards CAFs, after which the CAF phenotype is maintained via the mechanosensitive transcription factor YAP [44, 66]. In addition, the production of several cytokines (TGFβ, IL-6, CXCL12) by CAFs can contribute to the conversion of quiescent fibroblasts into CAFs [67–70]. These findings strongly suggest that CAFs could induce the conversion of other cells into CAFs in a positive feedback loop, which would explain the high abundance of CAFs found in solid tumors.

## CAFs and radiotherapy

**Fig. 1:**
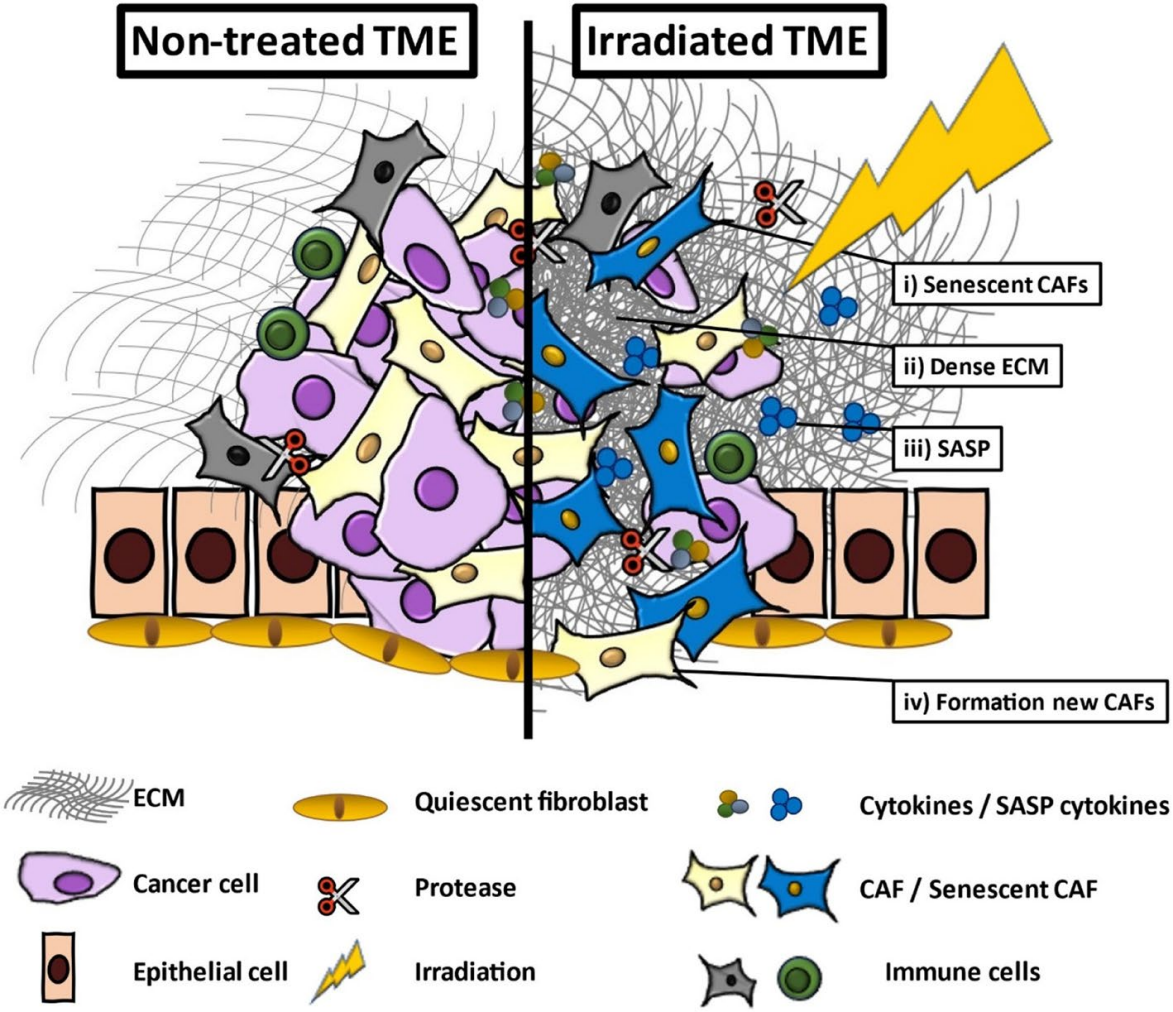
Radiation-induced changes in CAFs in the tumor micro-environment. Tumor cells are generally radiosensitive and thus eliminated by irradiation. In the stroma, i) CAFs persist in the TME in a senescent state, ii) the ECM becomes denser and stiffer, iii) the senescent CAFs display the SASP and iv) new CAFs are formed

The majority of cancer patients will be treated with radiotherapy as part of their treatment plan. Radiotherapy induces strong changes in the TME that affect the composition of the cellular landscape in the TME (Fig. 1). Vice versa, the composition of the TME also impacts the efficacy of radiotherapy, and CAFs play a major role in this process [2, 71].

### Tumor cell radioresistance by non-irradiated CAFs

Apart from contributing to tumor development, CAFs can also contribute to radioresistance of tumor cells. They can do this in various ways; via the secretion of soluble factors such as cytokines, growth factors and exosomes, via contact-mediated signaling, via modulation of the ECM, or via the induction of EMT or autophagy [2, 72]. For example, it has recently been shown that CAF-secreted IL-6 induces the growth and radioresistance of breast cancer cells both in vitro and in vivo by STAT3 phosphorylation [21]. Alternatively, CAFs contribute to tumor cell radioresistance via high SMAD3 expression, which can enhance the radioresistance of NSCLC cells via Akt signaling [73], or via the induction of NFkB signaling in nasopharyngeal carcinoma cells [74]. More indirectly, CAF-deposited collagen-1 can induce radioresistance by stimulating CXCL-1 signaling [75]. CAFs are thus able to induce radiotherapy resistance without being exposed to irradiation themselves.

### Irradiation impacts fibroblast phenotype

Radiotherapy can induce a pro-tumorigenic and pro-fibrotic phenotype in quiescent fibroblasts, as demonstrated in a study where epithelial cells gain tumorigenic potential when they are transplanted into an irradiated environment [76]. For CAFs, it has been shown that they are capable of surviving harsh conditions, including ablative radiotherapeutic doses up to 18Gy [77]. Rather than inducing cell death, ionizing radiation promotes a senescent CAF phenotype in for instance non-small-cell lung cancer (NSCLC), which affects CAF functioning in the TME [77]. These findings suggest that CAFs remain present in the TME after irradiation. As radiotherapy has been shown to induce significant changes in CAF phenotype with both pro- and anti-tumorigenic consequences [78, 79], this could be crucial for treatment outcome [58, 80].

### Irradiated CAFs retain many of their pro-tumorigenic features

After irradiation, the induction of a senescent CAF phenotype results in a pro-tumorigenic senescence-associated secretory phenotype (SASP), which has been associated with tumor growth, EMT and radioresistance [81]. In agreement, senescent fibroblasts express various proliferation-inducing factors (e.g. HGF, CXCL12 and EGF) [82, 83], can induce treatment resistance via inducing tumor cell EMT, or metabolically support tumor cell progression and metastasis by upregulating glycolysis and ketone production [28]. These findings suggest that CAFs with a radiation-induced senescent phenotype can contribute to tumor progression after radiotherapy, which would explain why targeting senescence-like fibroblasts is able to induce radiosensitivity in NSCLC cells [84].

More directly, irradiated CAFs have been shown to induce cancer cell EMT, via e.g. IL-6, IL-8 or exosome secretion after irradiation [53, 85, 86]. The resulting EMT enhances the radioresistance of cancer cells [87, 88]. In addition, radiotherapy can induce CAFs to increase their IGF-1 expression, which has been shown to contribute to reduce tumor cell radiosensitivity and may even contribute to metastasis [78]. Although reduced CAF-mediated immunosuppression after radiotherapy has been reported for specific immune cells [89, 90], it has been demonstrated that irradiated CAFs still possess immunosuppressive properties [58, 91], which could reduce the efficacy of combining radiotherapy with immunotherapy. In contrast, in rectal cancer, irradiation has been shown to induce an inflammatory, pro-tumorigenic phenotype in CAFs [92], demonstrating the complexity of the interaction between CAFs, immune cells and radiotherapy.

In conclusion, although irradiation induces numerous changes in CAF phenotype with anti- or pro-tumorigenic consequences [79], the majority of findings demonstrates that CAFs persist in the TME after irradiation and that irradiated CAFs tend to retain or even enhance their pro-tumorigenic characteristics. The induction of an irradiation-induced senescent phenotype of CAF is likely to contribute to this process.

### CAFs, ECM and radioresistance

The capacity of CAFs to reorganize the ECM, consisting of both matrix deposition and degradation, is affected by irradiation. The increased presence of matrix metalloproteases (MMPs) excreted by senescent CAFs can lead to EMT and induces a more stem cell-like phenotype in malignant cells [93]. Interestingly, the panel of proteases secreted by senescent cells strongly overlaps with the protease landscape found in malignant tumors [83], suggesting that MMP secretion by CAFs with an irradiation-induced senescent phenotype could contribute to tumor development in this manner.

The stiffness of the TME plays a large role in virtually all aspects of solid tumor progression, and also in radioresistance [94]. Despite radiotherapy transiently reducing interstitial pressure directly following treatment, it also induces excessive matrix deposition in which CAFs play a significant role [2] and which can decrease cancer cell therapy sensitivity [75, 95]. Increased CAF-mediated tissue stiffness after radiotherapy can generate a higher degree of hypoxic regions in the tumor by the compression of vasculature. The resulting hypoxia can induce CAF-like features in normal fibroblasts [96], and the resulting upregulation of HIF-1α in cancer cells contributes to cancer cell survival following radiotherapy [97, 98]. As radiation-induced ECM changes by CAFs also impair the permeation of possible neoadjuvant chemo- and/or immunotherapies, CAFs could not only contribute to radioresistance, but also to resistance against possible adjuvant therapy [99, 100].

Radiotherapy thus induces changes in the TME that affect cancer cell proliferation, immunogenicity, metabolism and ECM organization. As most radiotherapeutic treatment schedules are fractionated, the fact that irradiated CAFs could maintain or even gain properties that induce tumor cell radioresistance could strongly affect radiotherapy efficacy and should therefore be taken into account when treating patients with radiotherapy.

## The potential to adapt current treatments to prevent CAF-mediated therapy resistance

### Changing radiotherapy schemes to prevent CAF-induced radiotherapy resistance

In the last decades, increased precision in irradiation has enabled research into hypofractionated radiation schemes, in which patients are irradiated with higher dose per fraction, but with less fractions [101]. However, Hellevik et al. already established that single doses corresponding to either conventionally fractionated or hypofractionated radiation schemes do not induce apoptosis in CAFs derived from non-small-cell lung cancer (NSCLC) patients [77], suggesting that the pro-tumorigenic effects of CAFs are most likely not alleviated by changing the therapeutic radiation scheme. Therefore, other approaches to target the pro-tumorigenicity of CAFs in the irradiated TME are required.

### CAFs impact the efficacy of combination therapy

Radiotherapy routinely is not used as a stand-alone treatment, but in combination with chemo- and/or immunotherapeutic treatment. However, the presence of pro-tumorigenic irradiated CAFs in the TME potentially reduces the efficacy of combination therapy.

In the combination of radiotherapy and chemotherapy, the CAF-mediated collagen deposition that follows after radiotherapy impairs the delivery of chemotherapeutic compounds to the tumor [102, 103]. Reversibly, CAFs treated with chemotherapy promote tumorigenic and stem cell-like properties of NSCLCs cells, suggesting they can contribute to resistance towards both future chemo- and radiotherapy treatments [104]. In addition, radiotherapy-induced CAF exosomes activate the TGFβ pathway, which is associated with poor prognosis and resistance to both radiotherapy and chemotherapy in esophageal oral cancer (ESCC) patients [105].

CAFs are also likely to play a significant role in the efficacy of the combination of immunotherapy and radiotherapy. CAFs display strong immunosuppressive properties in the non-irradiated micro-environment, and sustain this phenotype upon irradiation [106, 107]. CAFs impair the anti-tumorigenic functioning of T cells, macrophages and dendritic cells and are therefore associated with low tumor immunogenicity [12, 80, 108], which suggests that they negatively affect the efficacy of combination radio-immunotherapy as well.

In conclusion, CAFs should not only be considered in all singular treatment modalities, but also in treatment plans that combine several types of therapeutic approaches.

### Targeting the CAF-induced pro-tumorigenic TME to modulate the immune response

By residing in the TME after irradiation, CAFs sustain the immunosuppressive milieu that prevents radiotherapy from eliciting an immune response against cancer cells. CAFs could thus impair the capability of dying cancer cells to act as ‘vaccines in situ’ and to elicit an anti-tumorigenic immune response that is able to target both the primary tumor site and distant metastases.

Therefore, treatment plans combining existing therapies with CAF-targeting approaches to generate an immunogenic microenvironment are currently being investigated. For example, targeting CAF-secreted CXCL12 by inhibiting its receptor CXCR4 synergizes with existing anti-PD-L1 therapy [12]. Most studies so far are combining CAF-based treatments with chemo- and/or immunotherapy. However, combining radiotherapy with CAF-targeting approaches holds strong promise for improving radiotherapy efficacy as well.

### Exploiting the ECM to target the CAF-induced pro-tumorigenic TME

Irradiated CAFs exert pro-tumorigenic effects by modulating the ECM. Therefore, targeting the functioning of CAFs by using ECM-based treatments could provide an attractive therapeutic strategy.

Proposed therapeutic approaches to modulate the ECM mostly aim to either inhibit the deposition of ECM components or to degrade the ECM that has already been formed. Approaches to soften the ECM have been shown to improve the penetration of chemotherapeutic agents and/or nanotherapeutics at the tumor site [109–111]. In addition, the decreased stiffness is able to facilitate vascular normalization via the decompression of blood vessels, resulting in reduced hypoxia [112]. Possibly, combining these approaches with radiotherapy can alleviate the negative effects of post-irradiation matrix stiffening on adjuvant therapeutic approaches.

Other methods to prevent post-irradiation matrix deposition mostly rely on anti-inflammatory and anti-oxidant approaches, which could also reduce either the immunogenic response or the DNA damage induced by radiotherapy [113]. Tissue stiffness should be seen as a delicate balance, as approaches to reduce stiffness could also have negative consequences for tumorigenesis.

It has been suggested that reducing ECM stiffness converts CAF from an αSMA^hi^FAP^lo^ phenotype towards an αSMA^lo^FAP^hi^ phenotype that is still pro-tumorigenic [114]. Such CAF phenotype plasticity possibly explains the difficulty of translating stroma-normalizing mechanotherapeutics towards the clinic [46, 115–117], as well as the increased tumor progression that is seen when depleting αSMA fibroblasts in a preclinical PDAC mouse model [14].

### Targeting pro-tumorigenic CAFs

The mentioned plasticity of CAF phenotype mainly concerns the interconversion of CAF phenotype between different activated CAF phenotypes. However, reverting CAFs towards a quiescent state could be a key factor to regain ECM homeostasis [118]. An overview of studies inducing the conversion of CAFs towards another phenotype using pharmaceutic compounds, including their main findings, can be found in Table 1. Although multiple studies demonstrate therapeutic potential for the induction of a quiescent phenotype in CAFs, more research is required to effectively implement these approaches in a clinical setting.

**Table 1:** Studies successfully targeting CAF phenotype using pharmacological approaches

Target	Approach	Tumor type	Combination	Stage	Result	Progression in clinic^A^	Ref
**ATM**	AZD0156, ATM inhibition	HNSCC, lung cancer	/	Preclinical	Reverse myofibroblast differentiation, increased CD8 infiltration	/	[18]
**Autophagy**	TR-PTX/HCQ-Lip, nanoparticles loaded with hydroxychloroquine and paclitaxel	PDAC	Paclitaxel (in the same NP)	Preclinical	Reduced αSMA/ECM, enhanced drug penetration	/	[110]
**Bet proteins**	JQ1, BET inhibitor	PDAC	Gemcitabine, interfering with DNA synthesis	Preclinical	Reduced CAF activation, reduced CAF-induced tumor cell proliferation, reduced TGFβ dependent gene expression, increased gemcitabine therapy efficacy	/	[119]
**BMP pathway**	Noggin, BMP signaling pathway inhibitor	Gastric cancer	/	In vitro	Reduced CAF markers: Reduced FAP, αSMA, Fibroblast-specific Protein (FSP), Thrombospondin 1 (TSP1), Vimentin	/	[120]
**Calcium ion channel**	Nifedipine, calcium channel blocker	Prostate cancer	/	Preclinical	Reduced tumor growth, normalization of CAF phenotype	FDA-approved	[121]
**CD36**	Sulfo-N-hydroxysuccinimidyl (SSO), CD36 inhibitor	HCC	/	In vitro + Preclinical	Reduced proliferation, reduced migration, reduced αSMA, FAP, Vimentin, IL-6, TGFβ, VEGFa. Reduced tumor growth in vivo	/	[122]
**CXCR4**	AMD3100, CXCR4 inhibitor	CRC	/	Preclinical	Reduced infiltration of αSMA + cells in the tumor, reduced metastatic development towards the liver	FDA-approved	[123]
	AMD3100, CXCR4 inhibitor	CRC	/	Preclinical	Reduced expression of αSMA, Vimentin, S100A4, FAP, reduced tumor growth	FDA-approved	[124]
**FGF19**	Fisogatinib, anti-FGF19 antibody	CRC	/	Preclinical	Reduced IL-1b production, reduced iCAF formation, reduced liver metastases	Phase I: NCT02508467	[125]
**HIF-1Α**	LNC-ACF, lipid nanoparticles loaded with acriflavine, inhibiting HIF-1α activation	Colorectal	LNC-PTX, lipid nanoparticles loaded with paclitaxel, inducing mitotic arrest and/or apoptosis	3D in vitro	Selective mortality in CAFs compared to tumor cells	Phase I: NCT00910728	[126]
**JAK**	AZD1480, JAK inhibitor	PDAC	/	Preclinical	Shift in CAF phenotype from iCAF to myCAF, decreased tumor growth	Phase I: NCT00910728	[9]
**JAK1/JAK2 + DNMT**	Ruxolitinib, JAK1/2 inhibitor + 5′-Azacytidine, inhibitor of DNA methylation,	Mammary carcinoma, head and neck	/	Preclinical	Loss of invasion-promoting and contracting phenotype	Ruxolitinib: FDA-approved Azacytidine: FDA-approved Combination: Phase II: NCT01787487	[4]
**KLF5-CXCL12**	Ketogenic diet	CRC	/	In vitro + preclinical	Reduced tumor growth, decreased CAF proliferation,	/	[127]
**Laminin-integrin interaction**	BM2, antibody blocking accessibility of laminin-332; A3IIF5, integrin a3 subunit blocking antibody	PDAC	/	In vitro	Reversion of differentiated CAFs into cells with normal-fibroblasts-like appearance	/	[128]
**Lipid synthesis**	Gold nanoparticles that inhibit MAPK, reverse EMT and increase lipogenic gene expression	PDAC	FASN inhibition	In vitro	Enhanced lipid synthesis, reduced αSMA	/	[129]
**NAMPT**	FK866, NAMPT inhibitor	CRC	/	In vitro	Reduced activation markers (αSMA, PDGFR) and stemness marker	Phase II: NCT00431912 Phase II: NCT00432107	[130]
**Non-defined**	sTRAIL-containing lipid-coated protamine DNA complexes	Bladder/PDAC	/	Preclinical	Delay of tumor growth, reversion towards quiescence	/	[131]
	Oxymatrine-loaded liposomes targeted towards tenascin C	Hepatocellular carcinoma	Lipid complex with icaritin and coix seed oil	Preclinical	Decreased αSMA, reduced collagen, EMT reversion, CAF deactivation, TAM M1 polarization	/	[132]
**NOX4**	GKT137831, NOX1/4 inhibitor	Lung	/	Preclinical	Reduced myofibroblast accumulation, reduced tumor growth	FDA-approved	[133]
**PDGFR**	Dasatinib, PDGFR inhibitor	Primary Lung carcinoma	/	In vitro	Reversion of primary ex vivo cultured CAFs towards a quiescent phenotype	FDA-approved	[134]
**PHD**	Dimethyloxalylglycine (DMOG), pan-PHD inhibitor	Lung	/	Preclinical	Reduced αSMA, reduced stiffness, reduced metastases	/	[135]
**Retinoic acid receptor**	Tamibarotene/AM80α	PDAC	Gemcitabine, interfering with DNA synthesis	Preclinical	Increased sensitivity to gemcitabine, CAFs display reduced αSMA, IL-6, collagen deposition	FDA-approved	[136]
	Tamibarotene/AM80α	UC + PDAC	a-PD-L1	Preclinical	Increased efficacy of a-PD-L1 therapy, polarization of macrophages towards M1, conversion of CAFs towards an anti-tumorigenic phenotype	FDA-approved	[16]
**Ros production**	nanoPue, aminoetyl anisamide-coated puerarin nanoemulsion	TNBC	a-PD-L1	Preclinical	CAF inactivation: Reduced αSMA/FAP/ECM deposition, reduced immunosuppressive phenotype, enhanced drug penetration	/	[111]
**RXFP1 (SMAD)** **TGFΒ pathway**	RLX-SPION, Relaxin-2 carrying nanoparticles	PDAC	Gemcitabine, interfering with DNA synthesis	Preclinical	Reduced hPSC activation: Reduced ECM deposition and angiogenesis, reduced tumor growth, increased chemotherapy efficacy	/	[137]
**Sigma receptor**	Retinoic acid-carrying minoethyl-coated mesoporous silica nanocarriers	Hepatoma	/	Preclinical	CAF normalization: Decreased αSMA, decreased FAP, decreased TME collagen production	/	[138]
**Sodium ion channel**	Flecainide, sodium channel blocker	Prostate cancer	/	Preclinical	Reduced tumor growth, normalization of CAF phenotype	FDA-approved	[121]
**TGFΒ**	Cyclophosphamide, TGFβ1 inhibitor	CRC	/	Preclinical	Reduced expression of αSMA, Vimentin, S100A4, FAP, reduced tumor growth and metastasis	FDA-approved	[124]
	Galunisertib	CRC	(5Z)−7-oxozeaenol	Preclinical	Decreased metastatic growth, decrease in fibronectin, reduced stroma recruitment in some cases	Phase II: NCT01246986	[139]
	CRE-NP(α-M), α-mangostin-loaded nanoparticles	PDAC	Gemcitabine, CRP-MC(trip)	Preclinical	CAF inactivation: Reduced αSMA/FAP/ECM deposition, enhanced chemotherapy efficacy	/	[109]
	Frax-NP-CGKRK, Fraxinellone-loaded nanoparticles	PDAC	siKras-LCP-ApoE3, nanoparticles to interfere with KRAS	Preclinical	CAF inactivation: Reduced αSMA/FAP/ECM deposition, enhanced drug penetration	/	[140]
	A83-01, TGFβ inhibitor	PDAC	/	Preclinical	Shift in CAF phenotype from myCAF to iCAF	/	[9]
	TGFβ-neutralizing antibody	Colorectal, melanoma	PD-1-targeting antibody	Preclinical	Loss of myCAFs and vCAFs, generation of ilCAFs, enhanced T cell infiltration, enhanced effect of PDL immunotherapy	/	[141]
	Triptolide, TGFβ inhibitor	PDAC	/	Preclinical	CAF inactivation: Reduced αSMA, fibronectin, collagen, hyaluronic acid, MMP9	/	[142]
**TGFΒ + TNFA**	Pirfenidone, anti-fibrotic agent + TGFβ inhibitor	Breast carcinoma	/	In vitro	Reduced CAF activation, reduced CAF migration	FDA-approved	[143]
	Pirfenidone, anti-fibrotic agent + TGFβ inhibitor	TNBC	Doxorubicin	Preclinical	Pirfenidone alone only reduces tumor fibrosis and TGFβ signaling, synergistic benefit on tumor growth and metastasis with doxorubicin	FDA-approved	[144]
**VDR**	Calcipotriol, VDR agonist	PDAC	Gemcitabine, interfering with DNA synthesis	Preclinical	Inhibited tumor progression, inflammation, fibrosis, enhanced drug delivery, reversion of PSC to quiescent state	Phase II: NCT00656019 *FDA-approved for topical application*	[118]
**Vita receptor**	All-trans retinoic acid (ATRA), vitA receptor agonist	PDAC	/	Preclinical	CAFs reverted back to quiescent state	FDA-approved	[145]
	ATRA/9-cis-RA	PDAC	/	In vitro	Reduced IL-6 production, reduced ECM production, reduced αSMA expression, reduced FAP expression	FDA-approved	[146]

*Abbreviations*: *ATM* Ataxia Telangiectasia Mutated kinase, *BET protein* Bromodomain and Extra-Terminal motif protein, *BMP protein* Bone Morphogenetic protein, *FGF19* Fibroblast Growth Factor 19, *HIF-1α* Hypoxia-Inducible Factor-1α, *JAK* Januse Kinase, *DNMT* DNA Methyl Transferase, *KLF5* Kruppel-Like Factor 5, *CXCL12* C-X-C motif Chemokine Ligand 12, *NAMPT* Nicotinamide Phosphoribosyltransferase, *NOX4* NADPH Oxidase 4, *PDGFR* Platelet-Derived Growth Factor Receptor, *PHD* Prolyl Hydroxylase, *RARα* Retinoic Acid Receptor-α, *ROS* Reactive Oxygen Species, *RXFP1* Relaxin Family Peptide Receptor 1, *sTRAIL* soluble Tumor Necrosis Factor (TNF) Related Apoptosis-Inducing Ligand, *TAM* Tumor-Associated Macrophage

^A^Progression in the clinic is provided as an indication of drug safety, not efficacy. Listed clinical trials and/or FDA approvals do not, per definition, refer to usage in an oncological setting

**Fig. 2:**
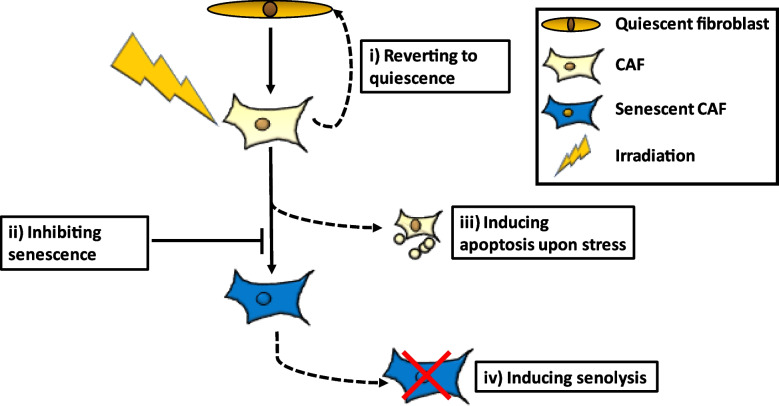
Therapeutic approaches to modulate CAF phenotype in a radiotherapeutic setting. Options include i) reverting CAFs towards a quiescent phenotype, ii) inhibiting the conversion towards senescence, iii) inducing apoptosis instead of senescence upon irradiation or iv) inducing cell death selectively in senescent CAFs.

As the radiotherapy-induced senescent phenotype of CAFs maintains or enhances the tumor-promoting characteristics of CAFs, modulating the conversion of CAFs towards senescent cells offers a promising strategy to interfere with the pro-tumorigenic CAF phenotype. Several approaches could prevent the presence of radiation-induced senescent CAFs in the TME, such as i) reverting CAFs towards quiescence, ii) inducing apoptosis instead of senescence upon stress, iii) inhibiting the conversion towards senescence or iv) inducing cell death of senescent CAFs (Fig. 2).

For example, the stimulation of vitamin D receptor (VDR) has been proposed as an approach to induce a more quiescent phenotype in CAFs in pancreatic cancer [118, 147], and inhibition of protein kinase C (PKC) signaling has been demonstrated to ‘steer’ fibroblasts towards apoptosis instead of senescence [148]. This suggests that, depending on the amount of genotoxic stress and the targeted protein, CAFs can be refrained from senescence upon irradiation, or undergo apotosis instead.

As another approach, the senescence of irradiated CAFs could be targeted directly by the usage of senolytics, drugs that are selectively lethal to senescent cells. For example, a selective inhibitor of the anti-apoptotic BCL-2 family members, Navitoclax (ABT-263), has been used to eliminate senescent astrocytes of irradiated glioblastomas [149]. Alternatively, FOXO4-DRI, a peptide inhibiting interaction between FOXO4 and p53, has been shown to target senescence-like fibroblasts in vivo, leading to improved NSCLC tumor radiosensitivity and reduced radiation-induced pulmonary fibrosis [84]. These findings suggest that neoadjuvant therapy with senolytic drugs could be used to eliminate pro-tumorigenic senescent CAFs from the irradiated TME.

Effective senolytic drugs may eliminate senescent CAFs from the TME following radiotherapy; they will however not target non-senescent CAFs or CAFs that are newly converted from quiescent fibroblasts. Therefore, additional approaches that selectively target all pro-tumorigenic CAFs could improve therapeutic potential.

### Selectively targeting CAFs

Selectively targeting tumor-promoting CAF subsets would present an attractive treatment modality to counteract CAF-mediated pro-tumorigenic effects. However, targeting drugs specifically towards CAFs poses significant challenges, as CAFs do not express exclusive CAF markers but are rather defined by a combination of markers and characteristics that are in itself not CAF-specific. Nevertheless, some markers allow for targeting CAFs more selectively. A selection of studies selectively depleting CAFs with pharmaceutics can be found in Table 2.

**Table 2:** Studies eradicating (subsets of) CAFs from the TME using pharmacological approaches

Target	Approach	Tumor type	Combination	Stage	Result	Ref
**ΑSMA**	Cellax, Docetaxel Conjugate Nanoparticles	Breast cancer	/	Preclinical	Reduction of αSMA^+^ stroma, reduced rigidity, enhanced tumor vascular permeability, reduced interstitial pressure, reduced metastasis	[112]
**FAP**	Pep-APCDs@Fe/DOX-LOS, nanoassembly that is activated by FAP activity	Breast cancer	/	Preclinical	Successful activation upon contact with CAFs, decreased fibrotic stroma, evoked strong antitumor immunity, and inhibited primary tumor and metastasis in the 4T1 tumor model	[150]
	Nano-clusters carrying ^1^O_2_-sensitive polymers, Ce6 and DOX	Breast cancer	Light irradiation	Preclinical	CAF depletion, suppression of tumor growth	[151]
	Single chain variable fragment-conjugated apoferritin nanoparticles (αFAP-Z@RT)	Breast cancer	PDT	Preclinical	Reduced tumor growth, induced immune response, CAF depletion	[152]
	ZnF_16_Pc-loaded ferritin nanoparticles	Breast cancer	PDT	Preclinical	CAF eradication, reduced level of collagen	[153]
	Antibody-photoabsorber conjugate	Colon adenocarcinoma	NIR-PIT	Preclinical	Suppressed tumor growth, higher CD8 + lymphocyte tumor infiltration, reduced αSMA + expression, activated host immunity	[154]
	FAP-targeting DNA vaccine	Colon and breast carcinoma	/	Preclinical	Reduced collagen type 1, higher drug infiltration, reduced tumor growth, reduced metastatic capacity	[102]
	PT630, inhibitor of FAP & DPPIV	Colon carcinoma	/	Preclinical	Decrease in tumor growth, excess collagen accumulation, reduced vascularization	[155]
	mAb F19, sibrotuzumab	Colorectal carcinoma, NSCLC	/	Clinical, phase I	Low toxicity, no objective tumor response	[115]
	FAP5-DM1, antibody linked to mitotic inhibitor	Lung, pancreas, head and neck	/	Preclinical	Long-lasting inhibition of tumor growth and complete regression	[156]
	αFAP-PE38, FAP-targeting Immunotoxin	Mammary carcinoma	/	Preclinical	Reduction of tumor growth, decreased recruitment of CAFs and TAMs, reduced angiogenesis	[157]
	DC-shA20-FAP-TRP2, Dendritic Cell Vaccine targeting FAP and TRP2	Melanoma	Dendritic Cell vaccination against tumor antigen	Preclinical	Reduced tumor growth (especially when co-targeting DCs to FAP and tumor antigen), increased immune cell infiltration and induction of anti-tumor immune response	[158]
	SynCon, microconsensus FAP DNA vaccine	Melanoma	TERT and PSMA tumor-targeting vaccines	Preclinical	Reduced tumor growth, synergistic effects with tumor-targeting vaccine approaches	[159]
	FAP-targeting CAR-T cells	Melanoma, colorectal, pancreatic, breast cancer	/	Preclinical	Limited or no tumor response, high toxicity (bone toxicity, cachexia)	[160]
	mAb F19, sibrotuzumab	Metastatic colorectal cancer	/	Clinical, phase II	No toxicity, minimal requirements for continuation of trial not met	[161]
	Targeted radionuclide therapy, ^177^Lu-FAP-2286	Metastatic pancreas, breast, rectum, ovary cancer	/	Clinical pilot study	Non-conclusive results on tumor growth, limited toxicity with acceptable side effects	[162]
	Chimeric DNA vaccine targeting FAP and survivin	Pancreatic cancer	Low-dose Gemcitabine	Preclinical	Successful induction of the immune response, reduced tumor growth, synergistic effects with gemcitabine	[163]
	CAR-T cells targeting FAP	PDAC	/	Preclinical	Reduced growth of PDX and syngeneic tumors, reduced ECM proteins, increased anti-tumor immunity	[164]
	CAR-T cells targeting FAP	NSCLC	/	Preclinical	Significant anti-tumor efficacy, enhanced antitumor immunity	[165]
	CAR-T cells targeting FAP	MPM	/	Clinical, phase I	Local delivery of CAR T cells targeting FAP is safe, not enough data to draw conclusions on efficacy	[166]
	UAMC-1110, small molecule FAP inhibitor	PDAC	Radiotherapy, anti-PD1	In vitro + preclinical	No effect on tumor growth, not in combination with radiotherapy or anti-PD1 therapy	[167]
	Antibody-photosensitizer	PDAC	tPDT	Preclinical	Increased detection of cleaved caspase 3, depletion of FAP-expressing stromal cells	[168]
	meso^FAP^ CAR-TEAM cells, expressing a mesothelin-targeted CAR and T-cell engaging molecule targeting FAP and CD3	PDAC	/	Preclinical + patient-derived organoids	CAF depletion, reduced tumor growth, increased survival, successful immune activation	[169]
	OMTX705, FAP-targeting antibody linked to cytolysin TAM470	PDX models of pancreatic, lung, breast and ovarian cancer	Gemcitabine, DNA synthesis inhibitor; Paclitaxel, inhibition of microtubule disassembly; Pembrolizumab, PD-1 inhibitor	Preclinical	Suppression of tumor growth in PDXs of several cancer types. Synergistic effect with gemcitabine, paclitaxel and PD-1 inhibition	[170]
	ValboroPro, FAP inhibitor	Previously treated metastatic colorectal cancer	/	Clinical, phase II	Significant, but incomplete inhibition of FAP enzymatic activity, minimal clinical activity	[171]
	Promelittin, FAP-activated protoxin	Prostate cancer	/	Preclinical	Reduction of tumor growth when injected intratumorally, high toxicity when injected intravascularly	[172]
	FAP-BiTE, bispecific T-cell engager targeting FAP and CD3	Prostate cancer	/	In vitro and Ex vivo	Depletion of CAFs, enhanced T cell activation and skewing of TAMs from M2 to M1, low toxicity	[173]
	FAP_PEP_-SLNP nanovaccine containing CpG adjuvant	Thymic Lymphoma, Colon Adenocarcinoma,	Doxorubicin, anti-PD-1	Preclinical	Reduced collagen density, reduced metastatic capacity, enhanced tumor drug uptake	[174]
	UCAR T-cells	TNBC	Mesothelin, anti-PD-1	Preclinical	Prolonged survival, reduced tumor burden, CAF depletion, reduced desmoplasia, enhanced lymphocyte tumor infiltration	[175]
**Fibronectin**	Doxorubicin-loaded hydroxyethyl starch – IR780 nanoparticles conjugated to CREKA peptide	Breast cancer	Light irradiation	Preclinical	Eredication of cancer stem cells, decreasing CAF population, reduced tumor growth, ECM degradation, reduced hypoxia	[176]
	CRE-NP9a-M, nanoparticles targeting fibronectin	PDAC	/	Preclinical	Successful targeting of CAFs, inactivation of CAFs, remodeling of the tumor microenvironment, enhanced blood perfusion	[109]
**FOXO4**	FOXO4-DRI, a peptide inhibiting interaction between FOXO4 and p53	NSCLC	Radiotherapy	Preclinical	Reduced radioresistance of NSCLC cells, lower incidence of radiation-induced pulmonary fibrosis	[84]
**GPR77**	Anti-GPR77 neutralizing antibody	Breast cancer	/	Preclinical	Eradication of CD10 + GPR77 + CAFs, abolishes establishment of clinical PDX, reverses chemoresistance of established PDX	[34]
**Non-defined**	Photothermal therapy + Gold nanoparticles (GIONF)	Desmoplastic cholangiocarcinoma	/	Preclinical	Depletion of αSMA^+^ stroma, reduced tumor stiffness, tumor regression	[177]
	Taxol (inhibitor of microtubule depolymerization)	Lung	5-fluoracil (thymidylate synthase inhibitor)	Preclinical	Reduced presence of αSMA^+^- CAFs, reduced drug resistance	[178]
	Disulfiram/copper	Nasopharyngeal carcinoma	/	Preclinical	Reduction in αSMA positive stroma, NPC cell and CAF apoptosis induction, increased necrosis	[179]
	Gemcitabine + Paclitaxel	PDAC	/	Clinical (ex vivo material)	Reduced αSMA + positive stroma, no difference in survival	[180]
	PEG5K-P(HEMASN38)x containing GDC-0449 + irinotecan	PDAC	/	Preclinical	Reduced αSMA, reduced collagen expression	[181]
	Polymeric micelle containing cyclopamine and paclitaxel	PDAC	/	Preclinical	Reduced αSMA + population, reduced FAP + CAF population	[182]
**Sigma receptor**	AEAA-MSNs@RA/GA, Aminoethyl anisamide-coated mesoporous silica nanocarriers	Hepatoma	/	Preclinical	Enhanced uptake of drug by CAFs, successful transformation into quiescent CAFs	[138]
**Smoothened**	IPI-926, Smoothened (SMO) inhibitor	PDAC	Gemcitabine, inhibition of DNA synthesis	Preclinical	Reduction in αSMA positive stroma with little effect on overall proliferation	[183]
**Tenascin C**	Nanoliposomes loaded with Navitoclax	Hepatocellular carcinoma	7pep-SSL-DOX	Preclinical	CAF depletion, reduced collagen, reduced tumor growth. Synergistic effect with DOX nanomedicine	[184]
	FH-SSL-NAV, Navitoclax-loaded nanoliposomes	Liver cancer	/	Preclinical	Reduced tumor growth, successful partial depletion of CAFs	[185]
	Liposomes containing captopril, ACE + SMAD inhibitor	PDAC	Gemcitabine-containing nanoliposomes	Preclinical	Reduced tumor growth, reduced ECM deposition, increased penetration of secondary treatment, reduced stroma	[186]

*Abbreviations*: *ACE* Angiotensin-Converting Enzyme, *DPPIV* Prolyl oligopeptidase family of serine proteases IV, *TRP-2* Tyrosinase-related protein 2, *TEAM* T-cell Engaging Molecule

Among the most promising markers to target CAFs selectively is fibroblast activation protein (FAP). FAP is upregulated in both non-senescent and senescent CAFs, with only relatively low expression on bone marrow mesenchymal stem cells [187, 188] FAP-activated prodrugs have shown promise in targeting CAFs and reducing tumor growth in multiple tumor models [150, 172], demonstrating that activated fibroblasts can relatively selectively be reached via FAP-targeting approaches.

Another characteristic of CAFs that could be exploited to target CAFs more specifically is the increased deposition of fibronectin on the CAF surface. Using synthetic peptides that bind to fibronectin, previous studies show that nanoparticles that are coated with these peptides display increased targeting towards the tumor, combined with an increased retention time [109, 189].

Although significant studies in autochthonous models or in the clinical setting have been conducted, many approaches targeting CAFs have primarily been explored in in vitro or in non-spontaneous xenograft models for various types of cancer (see Tables 1 and 2). While the majority of these studies reports positive effects as a result of selectively depleting CAFs, other studies suggest that depleting CAFs could have adverse effects on tumor progression and survival [14]. Preclinical xenograft or in vitro models certainly provide translational value, but are not able to accurately represent all interactions between tumor cells, stromal cells and immune cells present in spontaneously developed tumors. For example, the TME of xenograft tumors does not completely resemble the TME of autologous tumors, as both the stroma and the immune milieu differ substantially. Therefore, therapeutic approaches directly targeting CAFs warrant further research in different types of preclinical models.

## Conclusions

CAFs are key players in the TME. Despite the fact that CAFs have been ascribed several anti-tumorigenic functions, the majority of research indicates a pro-tumorigenic function for CAFs. CAFs affect the TME in several ways, via cell–cell contact, but also via their secretome, the modulation of the ECM and the immune milieu within the TME. In this way, they contribute to tumor initiation, progression and therapy resistance.

Upon radiotherapy, CAFs do not die but acquire a senescent phenotype. While sustaining the majority of their pro-tumorigenic function, such as their immunosuppressive capability and pro-survival signaling, they also acquire new properties. Irradiated CAFs strongly contribute to post-irradiation ECM reorganization, resulting in an increased TME stiffness, and they contribute to cancer cell radioresistance by inducing stem-cell like properties and the sustaining of a pro-tumorigenic TME.

Therefore, CAFs should be considered as key players in treated TME that are of vital importance for radiotherapy efficacy. In the future, CAFs may become targets for adjuvant therapy themselves. The modulation of CAF functioning before, during, or after therapy could possibly strongly improve treatment efficacy. Therefore, further research into CAF functioning in the irradiated tumor micro-environment is required, to mechanistically understand their role in therapy resistance and to design and/or adjust therapy accordingly.

**Table:** 

**Open key questions:**
- *What is the best strategy to target CAFs selectively?*
- *What are the possible adverse effects of targeting CAFs?*
- *How does radiotherapy change the CAF landscape and what does this mean for radiotherapy efficacy?*

## Data Availability

Not applicable.

## Abbreviations

BCL-2: B-cell lymphoma 2
CAF: Cancer-associated fibroblast
CXCL-1: C-X-C motif chemokine ligand 1
CXCL-12: C-X-C motif chemokine ligand 12
CXCR4: C-X-C chemokine receptor type 4
ECM: Extracellular matrix
EGF: Epidermal Growth Factor
EMT: Epithelial to Mesenchymal transition
ESCC: Esophageal oral cancer
FAP: Fibroblast activation protein
FOXO-4: Forkhead box protein O4
HGF: Hepatocyte growth factor
HIF-1α: Hypoxia-inducible factor 1 alpha
iCAF: Inflammatory cancer-associated fibroblast
IGF: Insulin-like growth factor
IL-10: Interleukin-10
IL-6: Interleukin-6
IL-8: Interleukin-8
MMP: Matrix metalloproteinase
myCAF: Myofibroblast cancer-associated fibroblast
NSCLC: Non-small-cell lung cancer
PDAC: Pancreatic ductal adenocarcioma
PD-L1: Programmed cell death ligand 1
PKC: Protein kinase C
SASP: Senescence-associated secretory phenotype
SMAD3: Mothers against decapentaplegic homolog 3
TAZ: Transcriptional co-activator with PDZ binding motif
TGFβ: Transforming growth factor β
TME: Tumor micro-environment
TWIST1: Twist-related protein 1
VDR: Vitamin D receptor
VEGF: Vascular endothelial growth factor
YAP: Yes-associated protein
αSMA: Alpha Smooth Muscle Actin
